# Supplementary material for: OVOL2-Mediated ZEB1 Downregulation May Prevent Promotion of Actinic Keratosis to Cutaneous Squamous Cell Carcinoma
Source: J Clin Med. 2020 Feb 25;9(3):618. doi: 10.3390/jcm9030618 (PMC7141138; doi:10.3390/jcm9030618)
Supplement: Supplementary file 1 [file jcm-09-00618-s001.pdf]

Supplementary Materials

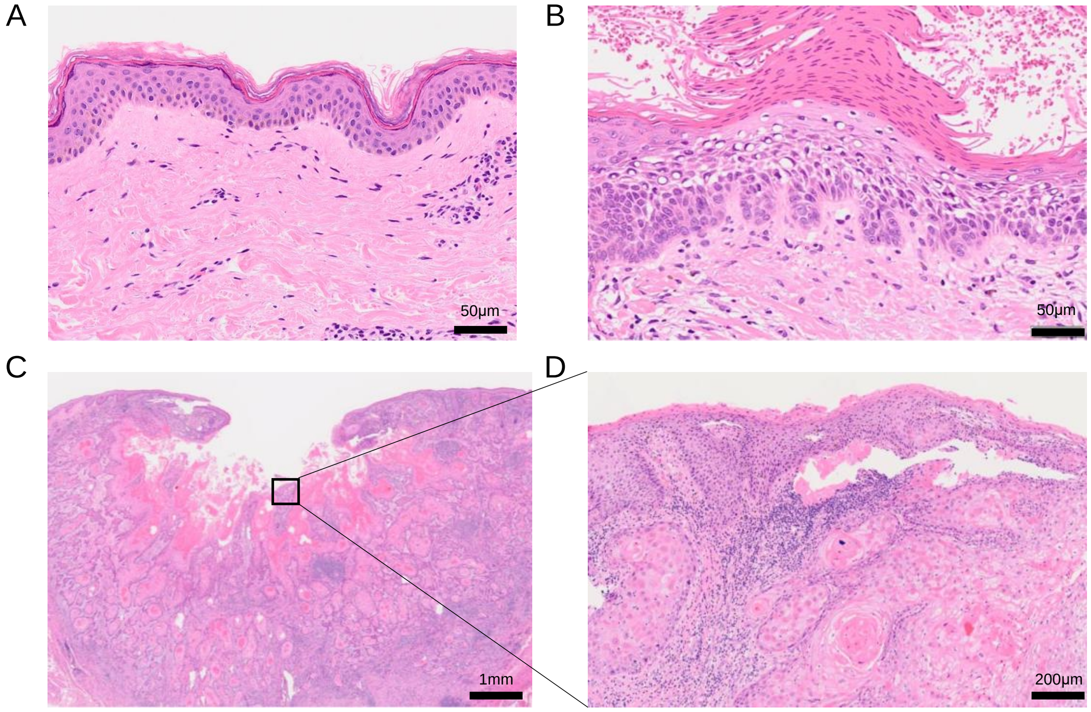

Supplementary Figure S1. Representative H&E images. (**A**) normal skin, (**B**) AK, (**C**) cSCC and (**D**) high-power view of cSCC in (**C**).

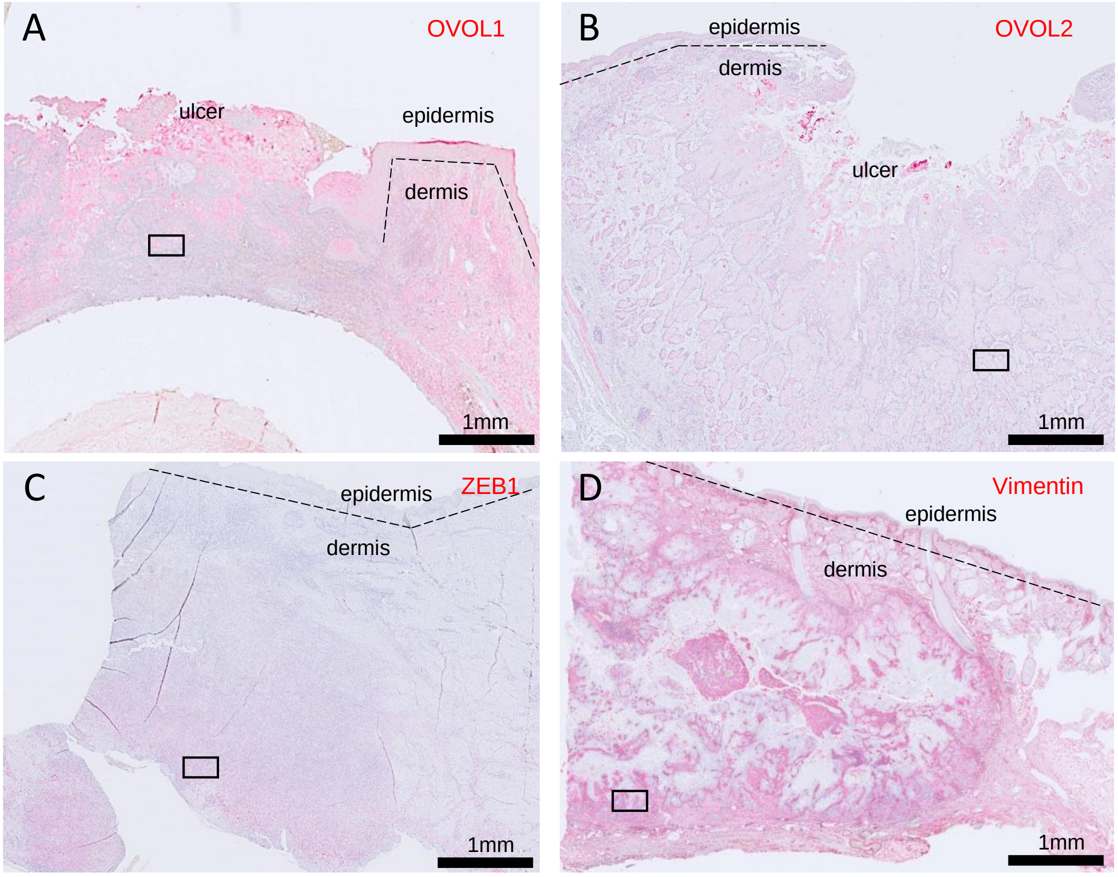

Supplementary Figure S2. Low-power view of cutaneous squamous cell carcinoma (cSCC) staining to show the location of tumor cells shown in Figure 1 (cSCC column). (**A**) OVOL1, (**B**) OVOL2, (**C**) ZEB1 and (**D**) vimentin. The framed rectangular areas were shown in Figure 1 (cSCC column).

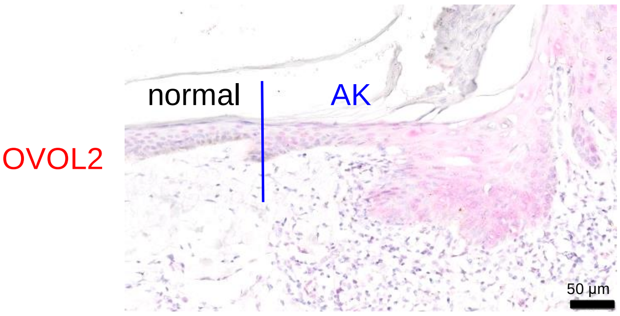

Supplementary Figure S3. Border between normal tissue and actinic keratosis (AK) stained for OVOL2.

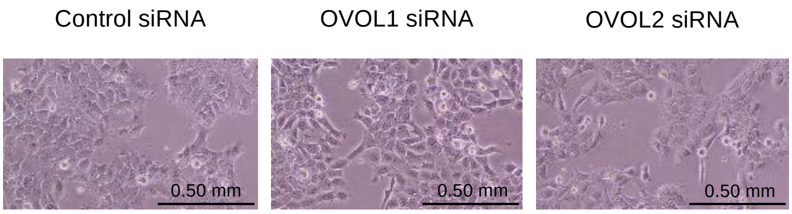

Supplementary Figure S4. Morphological features of A431 cells treated with control siRNA, OVOL1 siRNA, or OVOL2 siRNA 48 h post-transfection.

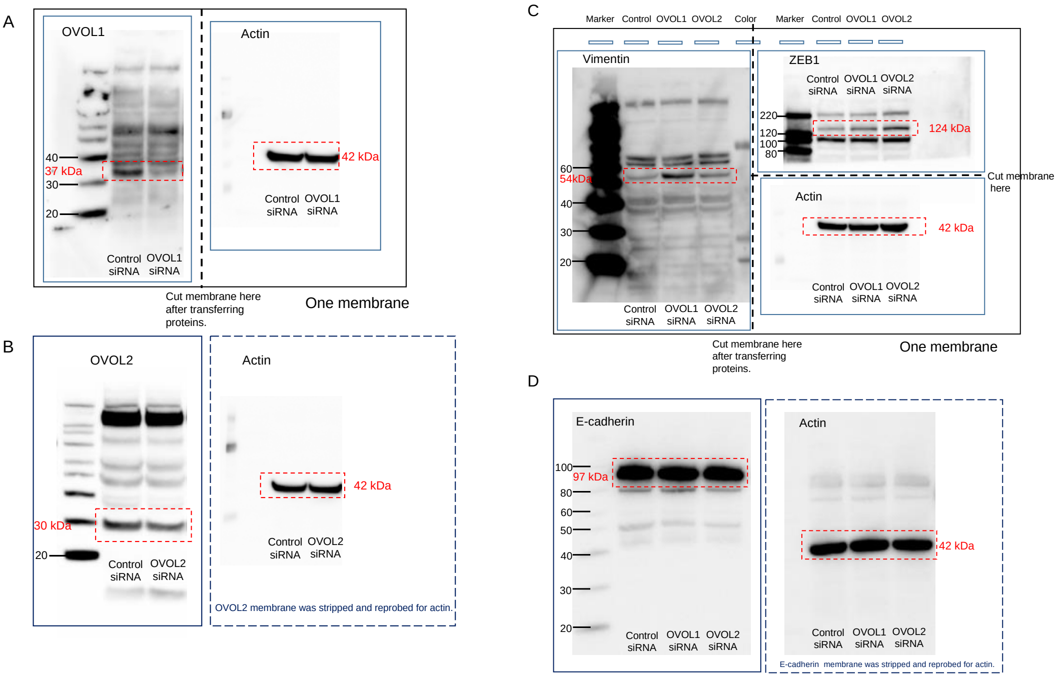

Supplementary Figure S5. Original images of western blots shown in Figure 3. Original western blot images (**A**) Figure 3A, left, (**B**) Figure 3A, right, (**C**) Figure 3B, left, and (**D**) Figure 3B, right.

Supplementary Table S1A. Associations between OVOL1/2 and ZEB1 expression in AK clinical samples

	**Total**	**OVOL1**		***P*-value**
		**High**	**Low**	
ZEB1				0.455
Positive	4	3	1	
Negative	26	23	3	
	**Total**	**OVOL2**		***P*-value**
		**High**	**Low**	
ZEB1				1.000
Positive	4	4	0	
Negative	26	21	5	

Fisher's exact test. *P*-values <0.05 were assumed to indicate a statistically significant difference.

Supplementary Table S1B. Associations between OVOL1/2 and ZEB1 expression in cSCC clinical samples

	**Total**	**OVOL1**		***P*-value**
		**High**	**Low**	
ZEB1				1.000
Positive	15	7	8	
Negative	15	6	9	
	**Total**	**OVOL2**		***P*-value**
		**High**	**Low**	
ZEB1				0.0604
Positive	15	3	12	
Negative	15	9	6	

Fisher's exact test. *P*-values <0.05 were assumed to indicate a statistically significant difference.

Supplementary Table S2A. Associations between OVOL1/2 and vimentin expression in AK clinical samples

	**Total**	**OVOL1**		***P*-value**
		**High**	**Low**	
Vimentin				1.000
Positive	3	3	0	
Negative	27	23	4	
	**Total**	**OVOL2**		***P*-value**
		**High**	**Low**	
Vimentin				0.434
Positive	3	2	1	
Negative	27	23	4	

Fisher's exact test. *P*-values <0.05 were assumed to indicate a statistically significant difference.

Supplementary Table S2B. Associations between OVOL1/2 and vimentin expression in cSCC clinical samples

	**Total**	**OVOL1**		***P*-value**
		**High**	**Low**	
Vimentin				1.000
Positive	12	5	7	
Negative	18	8	10	
	**Total**	**OVOL2**		***P*-value**
		**High**	**Low**	
Vimentin				0.458
Positive	12	6	6	
Negative	18	6	12	

Fisher's exact test. *P*-values <0.05 were assumed to indicate a statistically significant difference.
